# Clinical characteristics and long-term treatment outcomes of patients with new-onset epileptic seizures associated with COVID-19 infection

**DOI:** 10.3389/fneur.2026.1758330

**Published:** 2026-03-05

**Authors:** Fang Li, Lingqi Ye, Yuyu Yang, Wenjie Ming, Shuang Wang, Zhongjin Wang

**Affiliations:** 1 Department of Neurology and Epilepsy Center, Second Affiliated Hospital, Zhejiang University School of Medicine, Hangzhou, China; 2 Department of Neurology, Sir Run Run Shaw Hospital, Zhejiang University School of Medicine, Hangzhou, China

**Keywords:** anti-seizure medications, COVID-19, epilepsy, seizures, treatment outcomes

## Abstract

**Background:**

This study investigates the clinical characteristics and approximately two-year treatment outcomes of patients with new-onset epileptic seizures during the acute phase of COVID-19 infection.

**Methods:**

A retrospective, single-center cohort study was conducted from December 2022 to June 2023. The patients were categorized into two groups: those with acute encephalopathy (Group 1) and those without (Group 2).

**Results:**

This study enrolled a total of 34 patients (15 male and 19 female), with 18 assigned to Group 1 and 16 to Group 2. Patients in Group 2 (median: 32.5 years) were significantly younger than those in Group 1 (median: 60 years; *p* < 0.05). Status epilepticus was more frequent in Group 1 (66.7%, 12/18) compared to Group 2 (6.3%, 1/16; *p* < 0.001). Seizure latency was significantly shorter in Group 1 (median: 2 days) than in Group 2 (median: 9 days; *p* < 0.001). Abnormal posterior background activity on EEG was observed in 57.1% of Group 1 patients (4/7, *p* < 0.05), but in none of the Group 2 patients. However, a higher proportion of Group 2 patients showed interictal epileptiform discharges (72.7%, 8/11) compared to Group 1 (28.6%, 2/7). Epilepsy-related MRI abnormalities appeared in 22.2% (4/18) of Group 1 and 31.3% (5/16) of Group 2 patients. The proportion diagnosed with epilepsy was significantly higher in Group 2 compared to Group 1 (87.5% vs. 22.2%, *p* < 0.05). After 25 months of follow-up, one patient from each group developed drug-resistant epilepsy.

**Conclusion:**

New-onset epileptic seizures associated with COVID-19 generally have a favorable prognosis. A lower proportion of patients developed drug-resistant epilepsy.

## 1 Introduction

The Coronavirus Disease 2019 (COVID-19) pandemic, caused by severe acute respiratory syndrome coronavirus 2 (SARS-CoV-2), has led to a notable increase in neurological complications (1–3), including new-onset epileptic seizures. These seizures can occur in both individuals with pre-existing epilepsy and those without prior seizure history. Such cases, referred to as new-onset epileptic seizures following COVID-19 infection, have been documented in several studies (4–7). Within 6 months after SARS-CoV-2 infection, the cumulative incidence of new-onset seizures has been estimated at 0.81%, and of newly diagnosed epilepsy at 0.30% (8). Although these percentages may appear modest, when projected to the global population they correspond to millions of additional seizure events and hundreds of thousands of new epilepsy cases—a substantial public-health burden. Importantly, this elevated risk is not confined to hospitalized individuals: non-hospitalized COVID-19 patients also show a similarly increased incidence of seizures (9). Furthermore, new-onset seizures have been documented both in patients who develop COVID-19 associated acute encephalopathy (3, 10) and in those without any overt encephalopathy during the recovery phase of infection (11). Meanwhile acute encephalopathy is associated with worse outcomes in COVID-19 patients (2). Despite these observations, the long-term prognosis of COVID-19 related new-onset seizures remains poorly defined. In this study, we aim to compare the clinical features, electroencephalogram (EEG) findings, and longitudinal outcomes of new-onset seizures in COVID-19 patients with and without acute encephalopathy.

## 2 Methods

### 2.1 Patient selection and follow-up

We retrospectively reviewed the medical records of patients with new-onset epileptic seizures associated with COVID-19 who were admitted to our Epilepsy Center at the Second Affiliated Hospital, School of Medicine, Zhejiang University, between from December 1, 2022, to June 30, 2023. This study was approved by the institutional review boards of Second Affiliated Hospital of Zhejiang University. COVID-19 diagnosis was confirmed by a positive reverse transcription-polymerase chain reaction (RT-PCR) test or antigen test using nasopharyngeal swab samples (1). COVID-19 disease severity was categorized according to WHO guidelines as (1) non-severe: absence of viral pneumonia or hypoxia, or clinical signs of pneumonia but SpO_2_ > 90% on room air; (2) severe: clinical pneumonia with respiratory rate >30 breaths/min and SpO_2_ < 90% on room air; (3) critical: sepsis with organ dysfunction or requirement of mechanical ventilation (12). Patients with new-onset epileptic seizures were defined as individuals without a prior history of epilepsy who experienced seizures, including status epilepticus (SE), within 4 weeks of the onset of COVID-19 symptoms, a period termed the “acute phase” (13). Acute encephalopathy was defined as a rapidly developing (within 4 weeks) pathobiological brain process causing subsyndromal delirium, delirium, or coma (severely decreased consciousness), representing a significant change from baseline cognitive status (14). Patients were divided into two groups: Group 1 (patients with acute encephalopathy) and Group 2 (patients without acute encephalopathy).

Patients with a history of epilepsy, concurrent acute cerebrovascular events, brain trauma, or acute metabolic disturbances (such as electrolyte imbalance, hypoxemia, or hypoglycemia) were excluded. Follow-up assessments were conducted via telephone interviews or clinic visits, with a median follow-up of 25 months. Treatment outcomes were categorized into epilepsy, non-epilepsy, or unclassifiable. Patients diagnosed with epilepsy were further classified as having drug-resistant epilepsy (DRE) or non-DRE based on the International League Against Epilepsy (ILAE) consensus definition (15). Patients with seizures despite adequate trials of at least two well-selected antiseizure medications (ASMs) at appropriate dosages were classified as DRE.

### 2.2 Data collection

Clinical data were extracted from electronic medical records and epilepsy diaries. EEG recordings included either 24-h video EEG (VEEG) or routine EEG, following the international 10–20 electrode placement system. Non-contrast (native) brain MRI scans were performed for all patients. MRI acquisition followed routine clinical imaging protocols (including T1-weighted images, T2-weighted images, and fluid-attenuated inversion recovery sequence). Seizure latency was defined as the interval between COVID-19 symptom onset and the first seizure occurrence. Seizure types were classified based on clinical presentation and EEG findings according to the 2017 ILAE Classification system: focal aware seizures (FAS), focal impaired awareness seizures (FIAS), and focal to bilateral tonic–clonic seizures (FBTCS) (16). The categories of seizure frequencies include (17): (1) seizure clusters, defined as three or more seizures occurring within a 24-h period (18); (2) daily seizures; and (3) monthly seizures. EEG recordings were reviewed by an experienced electroencephalographer and categorized into: (1) abnormal posterior background rhythms (absence or slowing); (2) slow-wave activity, including diffuse slowing (intermittent or persistent) and focal slowing (intermittent or persistent); and (3) interictal epileptiform discharges (IEDs, focal or generalized). Imaging abnormalities on MRI were classified as normal, epilepsy-related abnormalities, or other abnormalities (those with an unclear relationship to epilepsy or incidental findings) (19).

### 2.3 Statistical analysis

Statistical analyses were performed using SPSS software version 25.0. Continuous variables were presented as median (interquartile range, IQR). Categorical variables were compared using Fisher’s exact test. Differences between groups for continuous variables were assessed using the Mann–Whitney U test. A *p*-value < 0.05 was considered statistically significant.

## 3 Results

### 3.1 Clinical characteristics

A total of 34 patients (15 male, 19 female) were enrolled in this study, and all participants were right-handed. The demographic and clinical characteristics are summarized in Table 1. Group 1 comprised 18 patients (10 male, 8 female) with a median age of 60 years (IQR: 31.0–74.5 years), including 13 severe / critical COVID-19 patients and 5 non-severe cases. Group 2 included 16 patients (5 male, 11 female) with a median age of 32.5 years (IQR: 17.0–39.0 years), all of whom were non-severe cases. A significant difference in age distribution was observed between the two groups (*p* < 0.05). There were no significant differences in education level and insurance coverage between the two groups of patients. No significant differences were found in medical history or history of febrile seizures. None of the patients had a history of alcohol consumption or a diagnosis of cognitive decline or dementia. During the acute phase, COVID-19 pneumonia was observed in 13 patients (72.2%, 13/18) in Group 1, while no patients in Group 2 experienced this complication. Five patients (27.8%, 5/18) in Group 1 required intensive care unit (ICU) admission. The median seizure latency was significantly shorter in Group 1 (2 days, IQR 1–4 days) compared to Group 2 (9 days, IQR 4–14 days; *p* < 0.001). The proportion of patients with SE was significantly higher in Group 1 (66.7%, 12/18) compared to Group 2 (6.3%, 1/16; *p* < 0.001). During the acute phase, in Group 1, seizure clusters were observed in 4 patients (22.2%, 4/18), while the remaining 14 patients (77.8%, 14/18) experienced daily seizures. The median duration of daily seizures was 3 days (IQR, 1–3 days), while the median duration of seizure clusters was 6 days (IQR, 5–12 days). In contrast, all patients in Group 2 (100%, 16/16) had monthly seizures. The median duration was 1 month (IQR, 1–4.75 months). The most common seizure type was FBTCS. In Group 1, lumbar puncture was performed on 15 patients, revealing increased intracranial pressure in 7 patients (46.7%, 7/15), pleocytosis in 4 patients (26.7%, 4/15), and elevated protein levels in 2 patients (13.3%, 2/15). No lumbar punctures were performed in Group 2.

**Table 1:** Clinical characteristics of the patients with new-onset epileptic seizures occurring during the acute phase of COVID-19 infection.

Variables	With acute encephalopathy (Group 1) (*n* = 18)	Without acute encephalopathy (Group 2) (*n* = 16)	*p*-value
Age at onset, years (median, IQR)	60 (31.0–74.5)	32.5 (17.0–39.0)	**0.007** ^**1**^
Sex, male (%)	10 (55.6)	5 (31.3)	0.154
Education			0.464
Lower	8	3	
Middle school	3	4	
Technical	3	4	
Higher	4	5	
Social health insurance type			0.759
URMI	7	8	
UEMI	9	6	
Other	2	2	
Previous history			NA
Chronic diseases^a^ (%)	8 (44.4)	0	
Traumatic brain injury (%)	2 (11.1)	0	
Cerebrovascular diseases (%)	2 (11.1)	1 (6.3)	
Brain tumor (%)	1 (5.6)	1 (6.3)	
Autism spectrum disorder (%)	0	1 (6.3)	
Febrile seizures (%)	1 (5.6)	2 (12.5)	NA
Associated neurologic condition			NA
Coma (%)	9 (50)	0	
Headache/dizziness (%)	3 (16.7)	2 (12.5)	
Sleep disturbance (%)	4 (22.2)	2 (12.5)	
Memory problem (%)	4 (22.2)	5 (31.3)	
Seizure latency^b^ (median, IQR)	2 (1–4)	9 (4–14)	**0.001** ^**1**^
COVID-19 pneumonia (%)	13 (72.2)	0	NA
COVID-19 disease severity			**0.000** ^**2**^
Non-severe (%)	5 (27.8)	16 (100)	
Severe + critical (%)	13 (72.2)	0 (0)	
ICU admission (%)	5 (27.8)	0	NA
Seizure frequency			NA
Seizure clusters (%) ^c^	4 (22.2)	0	
Daily (%) ^d^	14 (77.8)	0	
Monthly (%)	0	16 (100)	
Seizure classification			NA
FBTCS only (%)	16 (88.9)	12 (75)	
FAS/FIAS (%)	1 (5.6)	2 (12.5)	
FIAS/FAS + FBTCS (%)	1 (5.6)	2 (12.5)	
Status epilepticus (%)	12 (66.7)	1 (6.3)	**0.000** ^**2**^
CSF examinations (*n* = 15)			NA
Increased intracranial pressure (>180 mm H_2_O) (%)	7 (46.7)	–	
Pleocytosis^e^ (%)	4 (26.7)	–	
Protein elevation^f^ (%)	2 (13.3)	–	
Brain MRI			**0.017** ^**2**^
Normal (%)	5 (27.8)	10 (62.5)	
Epilepsy-related abnormalities (%)	4 (22.2)	5 (31.3)	
Other abnormalities^g^ (%)	9 (50.0)	1 (6.3)	
Number of ASMs			NA
None (%)	0	1 (6.3)	
1 (%)	8 (44.4)	11 (68.8)	
≥2 (%)	10 (55.6)	4 (25)	

IQR, interquartile range; ICU, intensive care unit; CSF, cerebrospinal fluid; FAS, focal aware seizures; FIAS, focal impaired awareness seizures; FBTCS, focal to bilateral tonic–clonic seizures; ASMs, antiseizure medications; UEMI, Urban Employees’ Medical Insurance; URMI, Urban Residents’ Medical Insurance; NA, not applicable.

^a^ Chronic diseases include coronary artery disease, hypertension, or diabetes mellitus; ^b^ Time interval from the initial manifestation of COVID-19 clinical symptoms to the occurrence of seizures; ^c & d^ occur only during the acute phase; ^e^ Pleocytosis refers to a white blood cell count of more than five cells per mm^3^; ^g^ Protein elevation is defined as >0.45 g/dL; ^e^ Other abnormalities include those with an unclear relationship to epilepsy or incidental findings.

1 Nonparametric Mann–Whitney U test.

2 Fisher’s exact test.Bold values indicate statistically significant differences (*P* < 0.05).

Group 1 showed a significantly lower rate of normal brain MRI results (27.8%, 5/18) compared to Group 2 (62.5%, 10/16; *p* < 0.05). Epilepsy-related abnormalities on brain MRI were observed in 22.2% (4/18) of patients in Group 1, including encephalomalacia and chronic infarcts, and in 31.3% (5/16) of patients in Group 2, including hippocampal sclerosis, other focal temporal lobe abnormalities, and tumors. Other abnormalities, such as white matter changes, acute inflammatory changes, and arachnoid cysts, were more prevalent in Group 1 (50%, 9/18) compared to Group 2 (6.3%, 1/16).

### 3.2 EEG characteristics

A total of 18 patients underwent VEEG recordings, including 7 patients from Group 1 and 11 patients from Group 2, and 6 patients underwent routine EEG recordings. VEEG findings are summarized in Table 2. Abnormal posterior background activity was observed significantly more frequently in Group 1 (57.1%, 4/7; *p* < 0.05), including absence (*n* = 2) and slowing (*n* = 2), whereas no such findings were seen in Group 2. Nearly half of the patients in Group 1 (42.9%, 3/7) had disrupted sleep architecture, while most patients in Group 2 (90.9%, 10/11) exhibited normal sleep architecture.

**Table 2:** VEEG findings in patients with new-onset epileptic seizures associated with COVID-19 infection.

Variable/Category	Group 1 (*n* = 7)	Group 2 (*n* = 11)	*p*-value
Posterior background			**0.011** ^**1**^
Normal (%)	3 (42.9)	11 (100)	
Abnormal (%)	4 (57.1)	0	
Sleep architecture			0.245
Normal (%)	4 (57.1)	10 (90.9)	
Abnormal (%)	3 (42.9)	1 (9.1)	
Slow wave			0.141
Normal (%)	1 (14.3)	4 (36.4)	
Intermittent slow activity (%)	4 (57.1)	7 (63.6)	
Persistent diffuse slow activity (%)	2 (28.6)	0 (0)	
Interictal epileptiform discharges			0.145
Absence (%)	5 (71.4)	3 (27.3)	
Focal (%)	2 (28.6)	7 (63.6)	
Focal & generalized (%)	0	1 (9.1)	
Ictal EEG pattern			NA
Not recorded (%)	5 (71.4)	10 (90.9)	
Recorded (%)	2 (28.6)	1 (9.1)	

VEEG, video electroencephalogram; EEG, electroencephalogram; NA, not applicable.

1 Fisher’s exact test.Bold values indicate statistically significant differences (*P* < 0.05).

**Figure 1:**
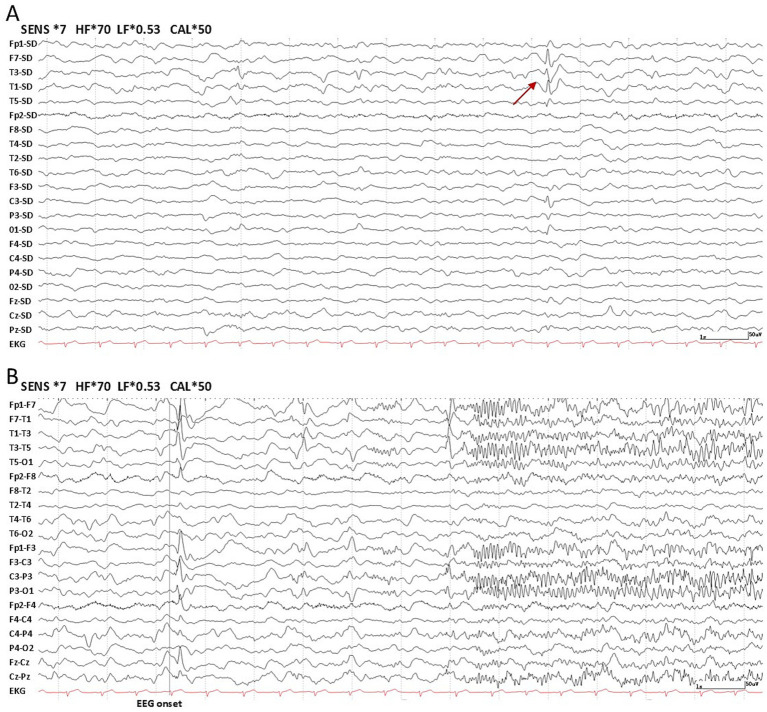
Laplacian montage and bipolar montage of scalp electroencephalographic (EEG) recordings for a patient experiencing acute encephalopathy in Group 1. Individual EEG channels are shown with an electrocardiographic recording at the bottom. Nomenclature is in accordance with the international 10–20 montage. Time scale: 15 mm/s, sensitivity: 7 μV/mm, low frequency filter: 0.53 Hz, high frequency filter: 70 Hz. **(A)** Focal sharp wave (maximum in T1, F7 and T3) in the left temporal region (interictal). The posterior background activity was absent. **(B)** Sharply contoured slow activity occurring in the left hemisphere, followed by the evolution of beta rhythm.

**Figure 2:**
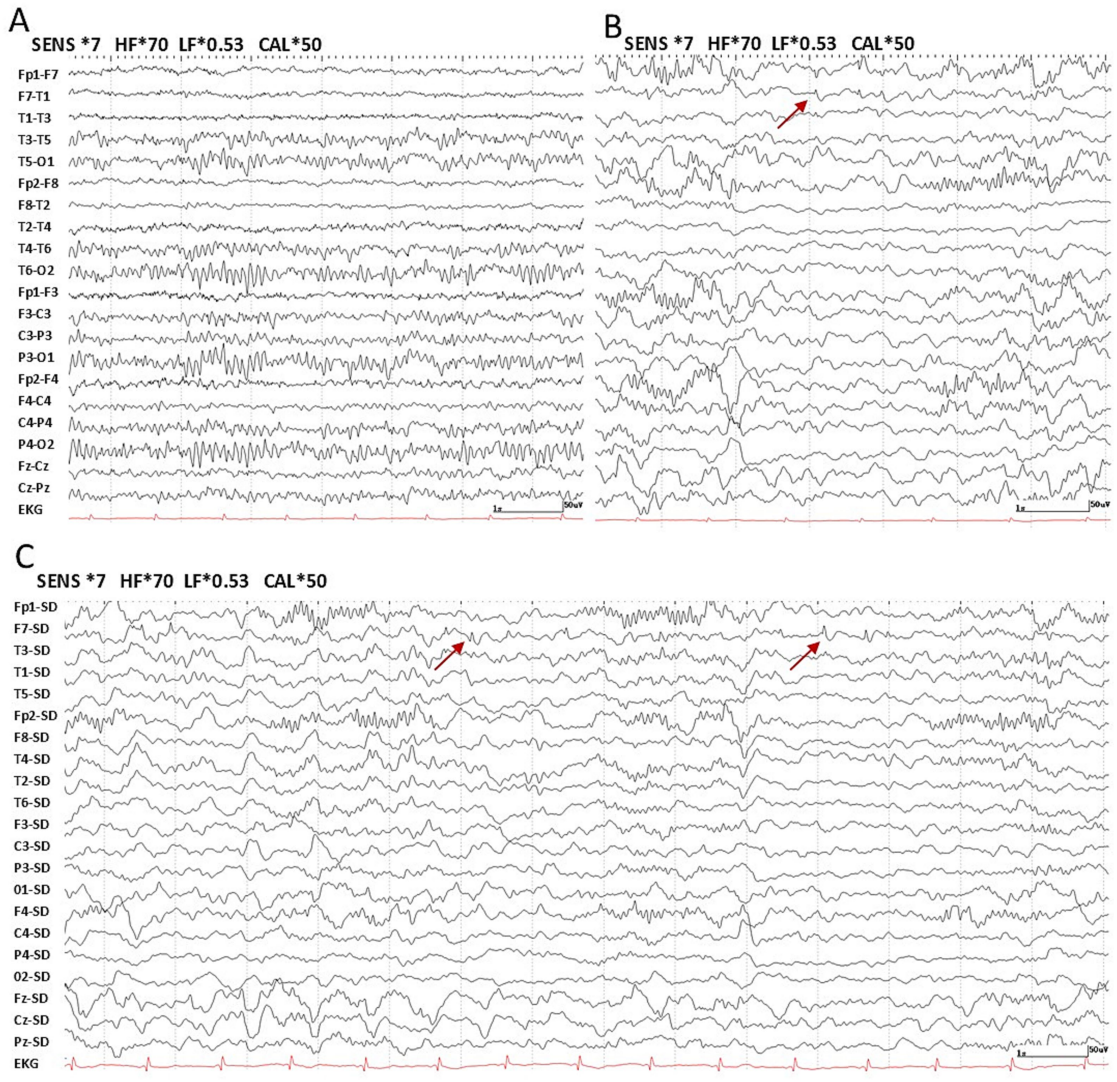
Laplacian montage and bipolar montage of scalp EEG recordings for a patient in Group 2. Nomenclature is in accordance with the international 10–20 montage. Time scale: 15 mm/s, sensitivity: 7 μV/mm, low frequency filter: 0.53 Hz, high frequency filter: 70 Hz. **(A)** Normal posterior background activity. **(B)** Focal sharp wave (maximum in F7) in the left frontal region (interictal) in the bipolar montage. **(C)** Focal sharp wave (maximum in F7) in the left frontal region (interictal) in the Laplacian montage.

Regarding interictal slow waves, persistent diffuse slow activity was observed in 28.6% (2/7) of Group 1, with no occurrences in Group 2. In contrast, intermittent slow activity was more frequent in Group 2 (63.6%, 7/11) than in Group 1 (57.1%, 4/7). Normal findings were noted in 14.3% of Group 1 and 36.4% of Group 2. IEDs were observed in 28.6% (2/7; Left: *n* = 1, Right: *n* = 1) of patients in Group 1, with one occurring in the frontal-central region and the other in the frontotemporal region (Figure 1A). In Group 2, 72.7% of patients (8/11; Left: *n* = 5, Right: *n* = 2, Bilateral: *n* = 1) exhibited IEDs, including four in the frontotemporal region, two in the frontal lobe (Figures 2B,C), and one in the temporal lobe. One patient in Group 2 showed multifocal discharges. Ictal recordings were obtained from two patients in Group 1 (Figure 1B) and one patient in Group 2.

### 3.3 Treatment outcomes

**Figure 3:**
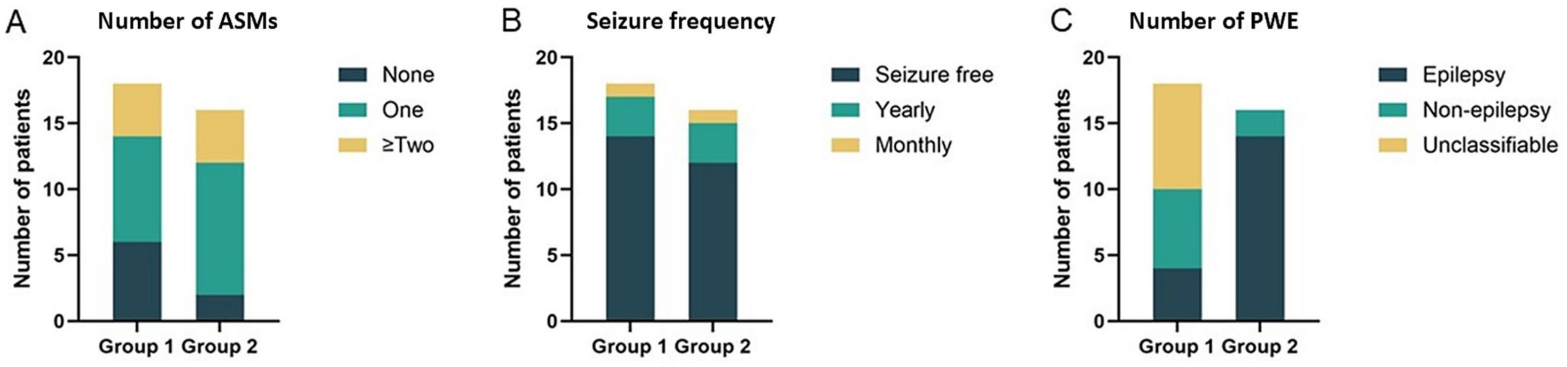
Treatment outcomes: **(A)** Number of anti-seizure medications maintained at last follow-up. **(B)** Seizure frequency during follow-up period. **(C)** Number of patients with epilepsy (PWE).

Overall, 76% of patients in this cohort (26/34) achieved seizure freedom after a median follow-up of 25 months. In Group 1, seizure freedom was achieved by 14 of 18 patients (77.8%, Figure 3B). The median time to achieve seizure freedom was 13 months (range 13–16 months). Among these, 6 patients (33.3%) successfully withdrew from ASMs within 6 months, 8 patients (44.4%) remained on a single ASM, and 4 patients (22.2%) were maintained on multiple ASMs (Figure 3A). In Group 2, seizure freedom was achieved by 12 of 16 patients (75%). The median time was 13 months (range 12–19 months). Among these, one patient experienced only a single seizure and did not require medication, while another remained seizure-free after discontinuing medication within 6 months. Four patients in Group 2 were treated with more than one ASM, while 10 patients were maintained on a single ASM. A significantly greater proportion of patients in Group 2 were ultimately diagnosed with epilepsy compared to Group 1 (87.5%, 14/16 vs. 22.2%, 4/18; *p* < 0.05) (Figure 3C). DRE occurred in one patient from each group.

## 4 Discussion

In this study, we retrospectively analyzed the clinical characteristics, EEG features, and treatment outcomes of 34 patients who experienced new-onset seizures following COVID-19 infection. Our findings suggest that new-onset epileptic seizures associated with COVID-19 generally have favorable outcomes, with few cases progressing to DRE. To our knowledge, although numerous case reports and systematic reviews regarding COVID-19-related seizures have been published previously (7, 11, 20–23), high-quality studies regarding the long-term prognosis of these patients with new-onset epileptic seizures remain scarce. This study, encompassing 34 cases of COVID-19-related seizures with a follow-up period of 25 months, addresses this knowledge gap and provides valuable insights for clinical management.

Patients with COVID-19-associated seizures and concurrent encephalopathy tended to be older and presented with more severe clinical manifestations, including higher incidences of SE and more frequent seizures, consistent with previous reports (3, 4, 22, 24). The latency period to seizure onset was significantly shorter in patients with encephalopathy (median: 2 days) compared to those without encephalopathy (median: 9 days), suggesting that older patients might experience more severe or rapidly progressive neurological injury following COVID-19 infection. Despite the advanced age and severe acute symptoms of patients with concurrent encephalopathy, only a small proportion ultimately developed epilepsy, and very few progressed to DRE, indicating an overall favorable prognosis. Therefore, it is essential to closely monitor elderly patients for early identification and aggressive management of status epilepticus to improve clinical outcomes.

EEG findings in Group 1 frequently included abnormal posterior background activity, disrupted sleep architecture, and persistent diffuse slow waves, which are features observed exclusively in this group. In contrast, IEDs were relatively uncommon, occurring in only 28.6% of cases. This aligns with existing literature (25, 26), which report that background abnormalities are the most common EEG finding in COVID-19 patients with altered mental status or seizure. The absence of reported sleep architecture disturbances in those studies is likely due to their inclusion of many patients undergoing routine EEG, rather than continuous VEEG. These results suggesting seizures in COVID-19 patients with encephalopathy likely arise from diffuse cortical dysfunction rather than localized cortical hyperexcitability. Takafumi Kubota et al. reported that 96.1% of COVID-19 patients undergoing EEG had abnormal background activity (26), significantly higher than the finding in this study. This discrepancy may result from their inclusion of a higher proportion of patients admitted to the ICU or those presenting with encephalopathy, who are more prone to EEG background abnormalities. Conversely, patients without encephalopathy exhibited a high prevalence of IEDs (72.7%), predominantly localized to the frontal and temporal regions, correlating closely with seizure occurrence. According to one study, 24-h EEG monitoring revealed IEDs in 92.1% of patients diagnosed with epilepsy who were not taking ASMs (27). This finding suggests that the higher positivity rate of IEDs observed in Group 2 aligns with the greater proportion of epilepsy cases within this group.

In Group 2, imaging was predominantly normal; however, epilepsy-related imaging abnormalities were detected in 31.3% of cases. This rate is higher than previously reported figures (18.7%) among newly diagnosed epilepsy patients of a similar age group (19), suggesting that COVID-19 infection may play a role in triggering seizures. Imaging abnormalities were more frequently observed in Group 1 (13 out of 18 patients), although these findings were primarily age-related rather than directly related to epilepsy.

However, our study has several limitations. First, the sample size was small, and the data were collected from a single center, which limits the generalizability of the findings. Age was considered a potential confounding factor in this study, and larger studies with more participants are needed to better delineate the effects of age on the outcomes. To enhance statistical validity and improve the robustness of the results, future research should include multicenter studies with larger sample sizes. Second, a longer follow-up period is needed to determine whether the patients will develop chronic epilepsy, which would help clarify long-term prognosis and the potential evolution of the disease. Third, due to the patients’ medical conditions and the national context during the COVID-19 pandemic, cognitive assessments and genetic testing were not feasible in this study. However, genetic and epigenetic factors are known to play a significant role in the onset and prognosis of epilepsy. Future studies that incorporate these factors could provide valuable insights into long-term recovery and prognosis.

## 5 Conclusion

New-onset epileptic seizures associated with COVID-19 generally have a favorable prognosis. A lower proportion of patients developed drug-resistant epilepsy.

## Data Availability

The dataset is restricted due to the inclusion of personal health information of patients. Requests to access the datasets should be directed to ZW, wzhongjin@zju.edu.cn.
